# Glycerol and Q-Tubes: Green Catalyst and Technique for Synthesis of Polyfunctionally Substituted Heteroaromatics and Anilines

**DOI:** 10.3390/molecules24091806

**Published:** 2019-05-10

**Authors:** Douaa Salman AlMarzouq, Noha M. Hilmy Elnagdi

**Affiliations:** 1 Department of Environmental Health, College of Health Sciences, the Public Authority of Applied Education and Training, P.O. Box 23167, Safat 13092, Kuwait; 2 Department of Organic Chemistry, Faculty of Pharmacy, Modern University for Technology and Information, Cairo, P.O. Box 12518, Cairo 11511, Egypt

**Keywords:** glycerol, green chemistry, modified Skraup’s quinoline synthesis, reactions under pressure, Q-tubes, polyaniline

## Abstract

The role of glycerol as a green bio-based solvent, reactant, and/or a catalyst in the synthesis of novel heterocycles, under pressure, is studied. Synthesis of novel quinolines in good yields using a new modified Skraup synthesis, utilizing glycerol and pressure Q-tubes, is demonstrated. Novel aniline trimers are prepared using glycerol, and substituted anilines under pressure, in acidic medium and water. Glycerol was employed as a catalyst and a green solvent in the synthesis of novel pyridazines **13a**–**c**. The mechanisms of the reactions and the catalytic effect of glycerol in protic and aprotic media are fully discussed. The structures of the synthesized compounds were determined via X-ray crystallography and spectroscopic methods.

## 1. Introduction

**Scheme 1:**
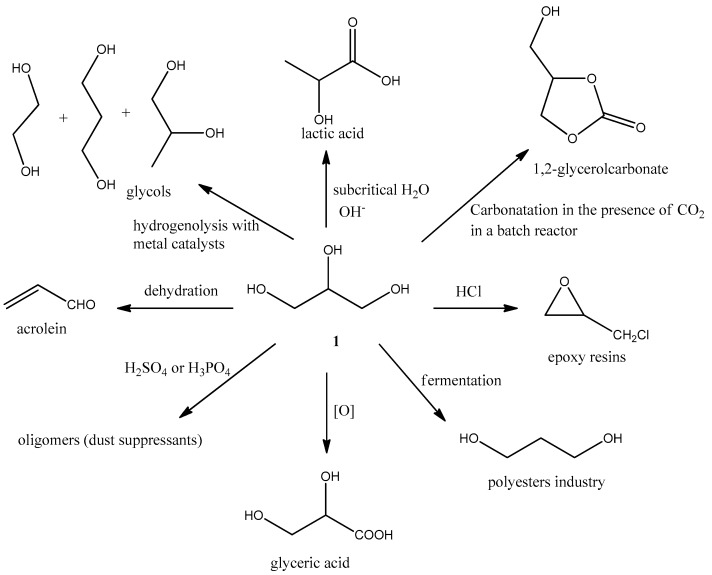
Some industrial uses of glycerol.

Glycerol was first isolated by the Swedish chemist: Carl W. Scheele, in 1779, upon treatment of olive oil with lead oxide [1,2]. Glycerol or propane-1,2,3-triol 1 is now produced in large quantities as a byproduct in many industries [3,4,5]. The large-scale production of biodiesel from fats, where glycerol is a waste product, has made glycerol a highly economic solvent and reactant [6,7,8,9,10,11,12]. This has attracted many researchers in the last two decades to find routes for converting this surplus into value added products [13,14,15,16,17,18,19,20,21,22,23,24]. Some of glycerol’s utilities in chemical industries are summarized in Scheme 1.

Since glycerol is highly hygroscopic and stable at high temperatures (B.P. = 290 °C), in addition to being environmentally friendly, nontoxic, nonvolatile, inflammable, non-corrosive, cheap, biodegradable, and recyclable, it could be considered a green medium and solvent in organic synthesis [25,26,27]. According to Arrhenius, the reaction rate doubles for every 10 °C rise in temperature [28]. Thus, glycerol would simply decrease the time of many reactions that take place in other low boiling-point solvents. Recently, Hamid et al. discussed the catalytic effect of glycerol by H-bond formation, which facilitates addition and condensation processes [29]. Glycerol has not yet been fully explored in the field of organic synthesis and catalysis. However, some studies have suggested that glycerol is a medium in “catalyst-free” reactions [30,31,32]. We think that these studies have neglected the possibility that glycerol itself acts as a catalyst in such reactions.

**Scheme 2:**
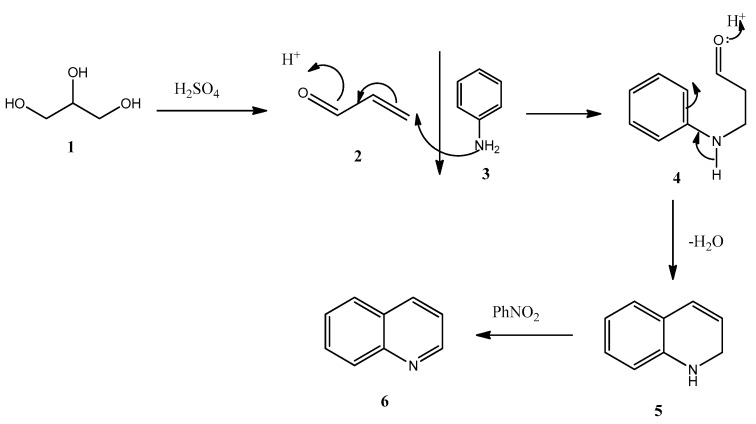
Reaction mechanism of the Skraup quinoline synthesis.

One of the oldest utilities of glycerol in organic chemistry is the synthesis of quinolines in poor yields, known as the “Skraup synthesis of quinolines” [33,34]. Skraup’s synthesis of quinolines has many limitations other than the low yield, including the multi-step addition of the reactants, in which glycerol must first react with H_2_SO_4_ to yield the hazardous acrolein (propenal), which is followed by the addition of the aromatic amine to form hydroquinoline that is converted to the quinoline by adding an oxidizing agent as the final step (cf. Scheme 2).

Other limitations include the use of highly concentrated glycerol that contains less than one-half percent of water known as “dynamite glycerin” to ensure good yields [35]. Precautions from explosion and physical hazards control have to be employed since the reaction is highly vigorous due to the use of nitrobenzene as an oxidizing agent and as a solvent. Prolonged heating in concentrated H_2_SO_4_, and the formation of a thick tar, from which quinoline is difficult to extract are also among the problems of the Skraup synthesis. These limitations have urged researchers to attempt to modify the synthetic route, to increase the yield of the quinoline synthesis reactions, and to make it “greener.” The new modified synthesis of quinolines or “green recipes” [36] involve using green solvents or even solvent-less reactions, one-pot synthesis, new green catalysts alongside with new technologies such as microwave energy, ultrasonication, and grinding [37,38,39,40,41,42,43,44,45,46,47,48,49].

There is now a boom in the use of high pressure in science [50], and reactions under high pressure and/or temperature proved to behave in a different manner than those under normal thermal conditions [51,52]. The newly invented borosilicate tubes known as Q-tubes have allowed the performance of difficult or otherwise impossible chemical reactions in chemical laboratories [53,54,55,56]. Shifts in transition state along a reaction coordinate, a switch of the rate-determining step, and the possible transformation of a transition state into a stable minimum, are among the possible phenomena that can occur for reactions under pressure, especially in fluids [57]. A supercritical solvent is a solvent that is subjected to a temperature and pressure higher than those of its critical point. However, when both the temperature and/or the pressure are lower than those of the critical point, and the temperature is higher than that of the boiling point, with a pressure higher than 1 bar, a subcritical solvent is obtained. A subcritical solvent can be defined as a hot compressed solvent and, according to Galy et al., glycerol produces different products in subcritical and supercritical solvents [58]. In this study, we combined glycerol and water as green, efficient solvents, in addition to the pressure in Q-tubes, to modify the synthesis of quinolines and prepare new aniline trimers and pyridazines.

## 2. Results and Discussion

We initially started our work with the synthesis of quinolines. Quinolines are extremely important in the pharmaceutical industry, especially in the treatment of malaria and cancer [59,60,61]. Fluoroquinolines are used in many pharmaceutical compounds, especially in fluoroquinolone antibiotics, such as ciprofloxacin (Cipro), gemifloxacin (Factive), levofloxacin (Levaquin), moxifloxacin (Avelox), norfloxacin (Noroxin), and ofloxacin (Floxin) [62,63]. 

**Scheme 3:**
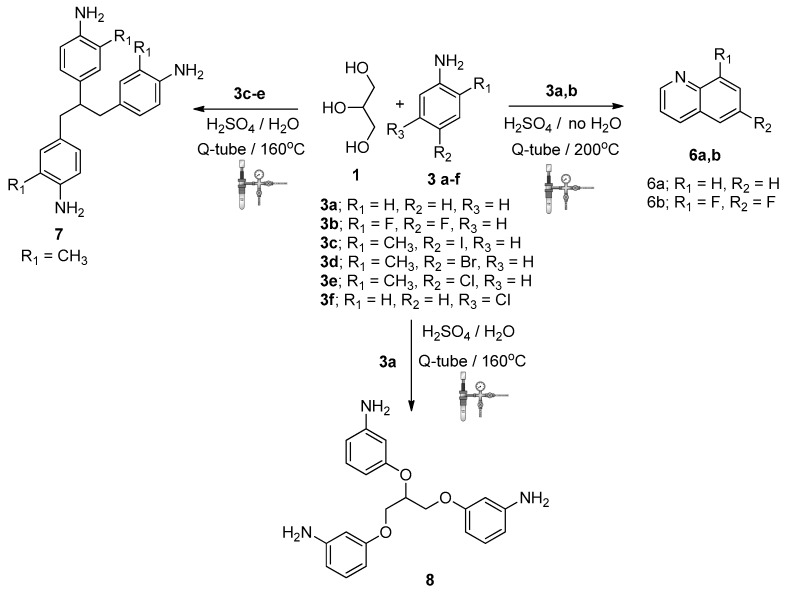
Reaction of aromatic amines, glycerol, and sulfuric acid in the presence/absence of water, under a high pressure, and high temperatures in the Q-tubes.

Selivanova et al. found that reacting polyfluoro-2-naphthylamines with glycerol in H_2_SO_4_ or CF_3_SO_3_H at 150–160 °C gives, surprisingly, the respective polyfluorobenzo[f]quinolones, rather than the expected cyclization at the unsubstituted ortho-position [39]. We thought of investigating this phenomenon as well as modifying the Skraup quinoline synthesis using pressure Q-tubes, and studying the effect of glycerol in subcritical and supercritical solvents. Unlike the results reported by Selivanova et al., the reaction of **1** and **3a**–**b** in the presence of conc. H_2_SO_4_, under pressure in a Q-tube at 200 °C proceeded to yield quinolines **6a** and **6b**, in 58% and 60% yield, respectively. To our knowledge, quinoline **6b** has not been previously isolated (cf. Scheme 3).

Recently, Saggadi and co-workers synthesized 5-substituted, 6-substituted, 7-subsituted, and 8-substituted quinolines using microwave conditions, aniline derivatives, and glycerol in the presence of sulfuric acid and water. The desired quinolines were obtained in 10%–66% yields [64]. Surprisingly, in our hands, the reaction of aromatic amines, glycerol, sulfuric acid, and water in pressurized conditions afforded a compound with an *m*/*z* value of 359.23, which was, after other spectroscopic investigations, assigned to 4,4′,4″-(propane-1,2,3-triyl)tris(2-methylaniline) 7. The ^1^H NMR spectrum of compound 7 shows a singlet at δ = 2.00 ppm for 9H that is assigned to 3-CH_3_ protons, a multiplet at δ = 3.34 ppm for 5H of 2 methylene and 1 methine protons, and another singlet at δ = 5.01 ppm for 6H assigned for the 3 NH_2_ protons as well as a multiplet at δ = 7.13 for aromatic 6 protons. The ^13^C-NMR spectrum of compound 7 shows a signal at δ = 146.53 assigned for 3C-NH_2_ at δ = 76.24 for 2CH_2_ and at δ = 16.95 for 3CH_3_. Conversely, when the unsubstituted aniline 3a was reacted under the same conditions, with glycerol and water under pressure (subcritical water), the aniline trimer 8 was the only product obtained. The structure of compound 8 was also confirmed via spectroscopic analysis. The mass spectrum of the reaction product 8 showed a molecular ion peak: *m*/*z* = 365.173 (100%). The ^1^H NMR spectrum revealed a singlet at δ = 2.4 ppm, integrated for 6H, which was assigned to the 3NH_2_ protons. Two doublets at δ = 3.27 ppm and 3.29 ppm for two aliphatic CH_2_ groups and multiplet at δ = 3.35 ppm were assigned for the methine CH proton and another multiplet at 6.94 ppm were assigned for the aromatic protons. The ^13^C-NMR spectral results were in agreement with the proposed structure, which showed a signal at δ = 63.09 ppm, assigned to the 2 O-CH_2_ carbons, as well as another signal at δ = 72.49 ppm, assigned to the O-CH carbon. It is worth mentioning that trials to use 3f as the starting aniline produced a compound with an *m*/*z* value of 410.5, for which we could not assign any reasonable, suggested structure. Table 1 summarizes our results and reaction conditions of the reactions of glycerol with aromatic amines under pressure.

**Scheme 4:**
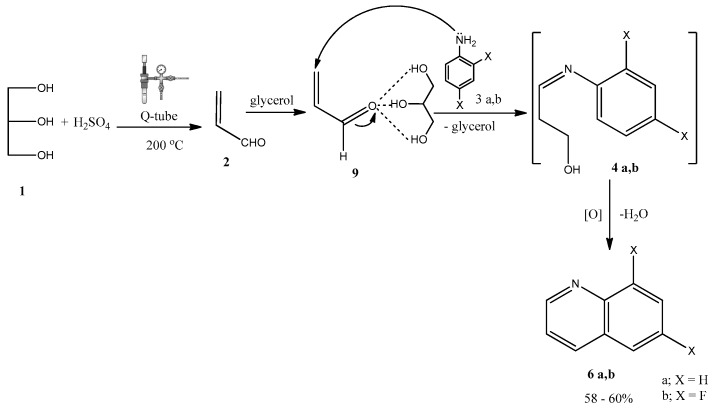
Mechanism of our modified-Skraup’s synthesis of quinolines under high pressure using Q-tubes.

While acrolein is a minor product of the dehydration of glycerol under neutral hydrothermal conditions, it becomes the main product when an acid catalyst is added, but at temperatures above 340 °C using conventional heating [58]. Under pressure, we could achieve the same results but at much lower temperatures. Thus, using Q-tubes and heating at only 200 °C for 1 h, we could prepare quinolines in high yields. Recently, the catalytic role of glycerol via H-bond was published by Hamid and coworkers [29]. We think that, in our work, the reaction proceeds via a typical Skraup reaction mechanism, but with glycerol having a dual role where one mol of glycerol acts as a starting material reacting with H_2_SO_4_ to produce acrolein **2**, and another mol of glycerol acts as a catalyst and bounds via H-bond with acroline to generate complex **9**. This then undergoes a Michael addition with the aromatic amines (**3a, b**). This is followed by the release of glycerol once more to produce the intermediate **4a, b** that then cyclizes to **6a** and **6b**, respectively. Scheme 4 shows a suggested reaction mechanism for the formation of quinoline **6a, b**. In our reaction conditions, there were no need to add any oxidizing agents since we believe that concentrated H_2_SO_4_ acts as a condensation agent and an oxidant.

**Figure 1:**
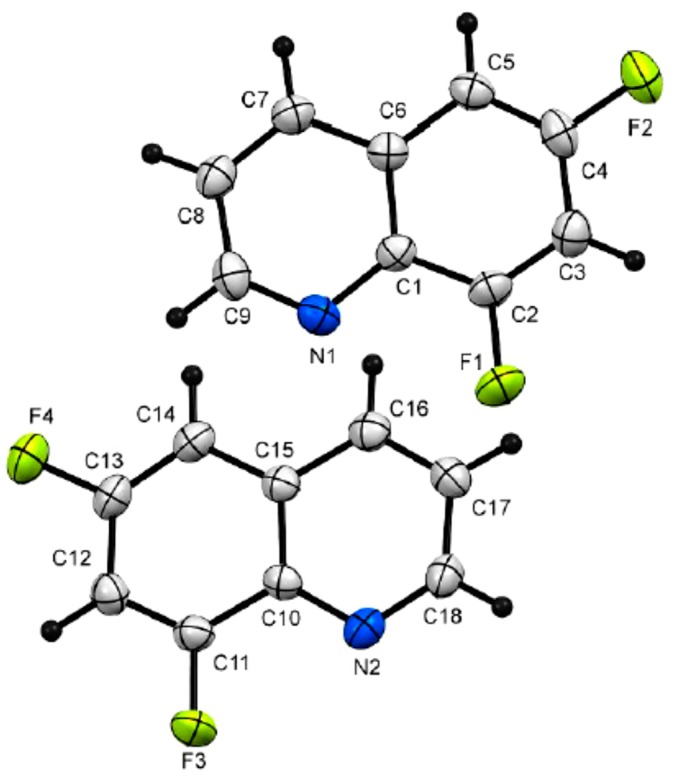
X-ray crystal structure of 6,8-difluoroquinoline **6b**.

**Figure 2:**
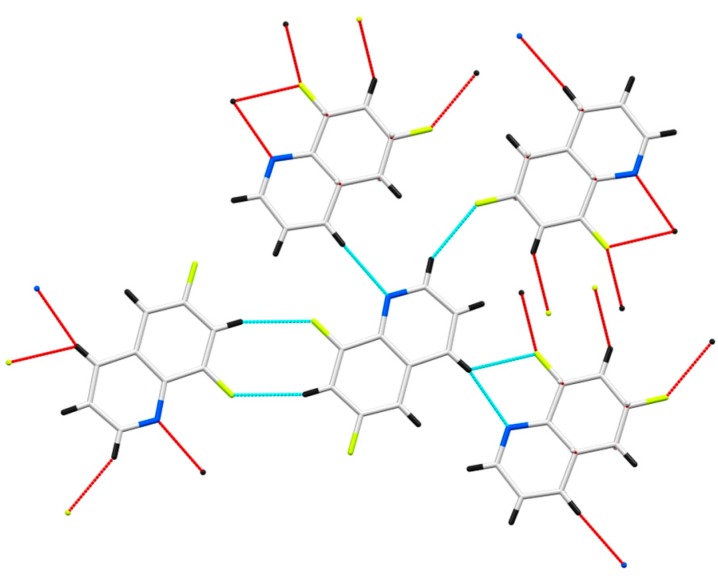
Aggregates of 6,8-difluoroquinoline **6b** that shows H-7 and F-8 H-bonding.

A member of our group investigated the x-ray structure of compound 6b, which confirmed the suggested structure, and no loss of fluorine atoms occurred under our reaction conditions [65] (Cf. Figure 1). The preliminary inspection of the X-ray crystallographic data of 6(b) indicated that the molecules exist in aggregates, via intermolecular H-bonding between Fluorine at C-8 and the hydrogen at C-8, as well as between N-1 and H at C-4 (Cf. Figure 2). A detailed discussion of the x-ray structure of these quinolines will be reported separately after exploring this phenomenon with other compounds.

**Scheme 5:**
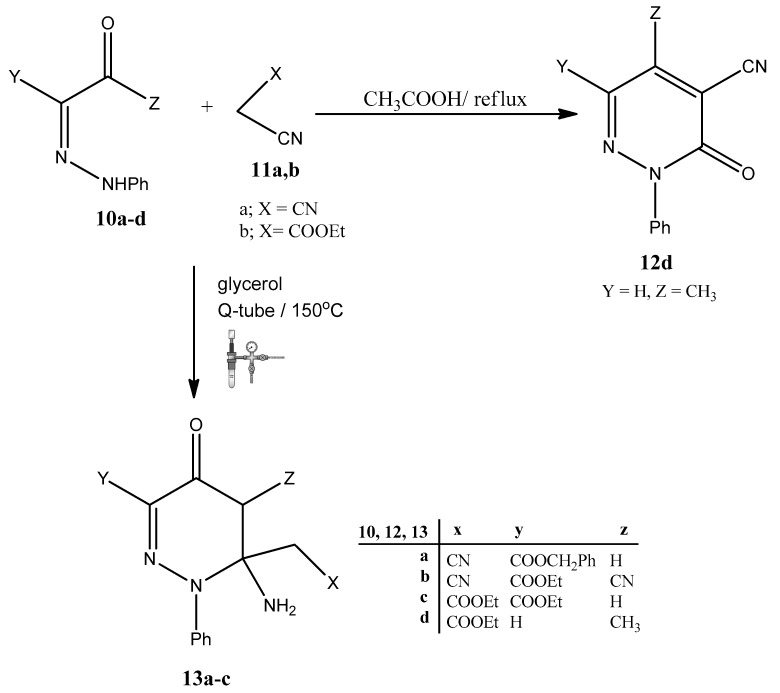
Synthesis of 4-oxo-1-phenyl-1,4,5,6-tetrahydropyridazine-3-carboxylate **13a–c**.

Subsequently, we shifted to utilizing glycerol in the synthesis of pyridazines. The pyridazine ring is an important structural feature in a number of pharmaceutical compounds, such as hydralazine (brand name Apresoline, vasodilator, US, FDA), cefozopran (anti-bacterial agent, Japan), and pipofezine (brand name Azafen or Azaphen, antidepressant, STADA, Nizhny Novgorod, Russia). Pyridazine derivatives have been reported to possess various pharmacological activities and intermediates for drugs synthesis, including antimicrobial, analgesic, anticancer, antitubercular, antidiabetic, antifungal, antihypertensive, anticonvulsant, anti-HIV, antiasthma, anti-inflammatory, phosphodiesterase (PDE) inhibitors, cyclooxygenase (COX) inhibitors, antipyretic, insecticidal, and neurological [66,67,68,69,70,71,72,73]. The reaction of arylazo **10d** and ethylacetoacetate **11b** in either acetic acid or ethanolic KOH, followed by reflux to afford pyridazinones **15d**, has been reported. This reaction was limited to the formation of **12d**, and was not consistently successful and Trials to form **12a** failed, since malononitrile dimerized under these conditions [74,75]. In this case, we could efficiently prepare pyridazinones **13a**–**c** in good yields, by the reaction of malononitrile **11a**, phenylhydrazono esters **10a**–**c**, and glycerol, either under conventional heating at 250 °C for 5 h, or under pressure in the Q-tubes at 150 °C for 30 min (cf. Scheme 5).

It was found that the yield of formation of **13a**–**c** increased significantly under reactions caused by pressure, Table 2 summarizes our findings and compares these reaction yields.

**Scheme 6:**
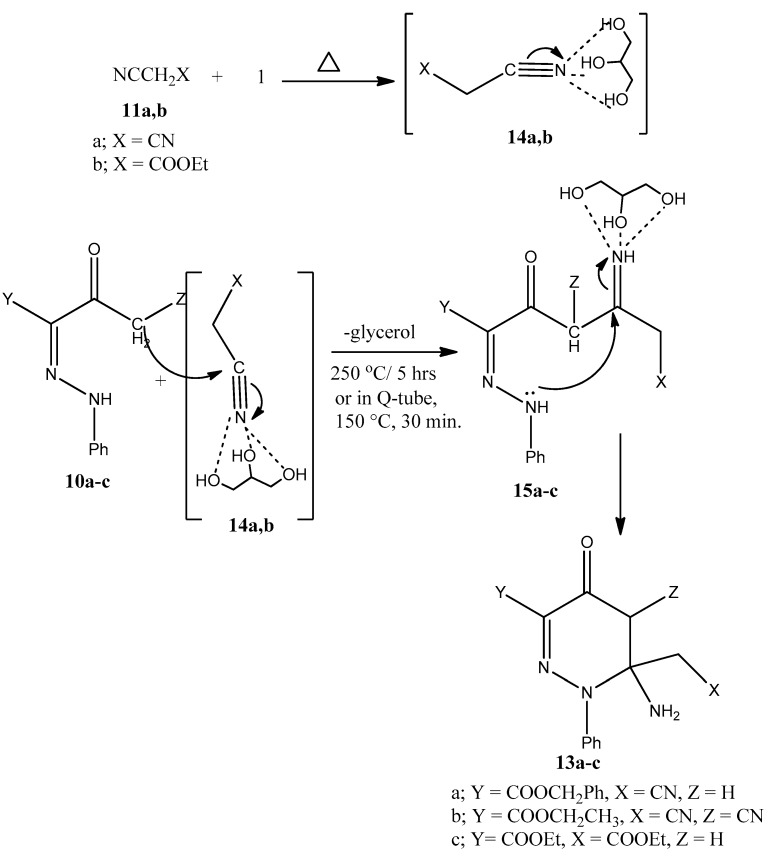
Mechanism of synthesis of 4-oxo-1-phenyl-1,4,5,6-tetrahydropyridazine-3-carboxylate **13a**–**c**.

We assume that glycerol acts as a catalyst and a bio-based solvent. The catalytic activity of glycerol in this reaction might be via H-bond formation with the N-atom in compound **11**, to form the intermediate **14**. The H-bond formation facilitates the Michael-type addition of the active methylene of the arylazo **10** on the even more electron-poor CN carbon forming the protonated imine **15**. It is suggested that another mole of glycerol coordinates with protonated imines **15**, followed by an intramolecular cyclization and glycerol is released again to the medium, which affords the 4-oxo-1-phenyl-1,4,5,6-tetrahydropyridazine-3-carboxylate**13a**–**c.** Scheme 6 shows our suggested mechanism for the formation of **13a**–**c**.

**Figure 3:**
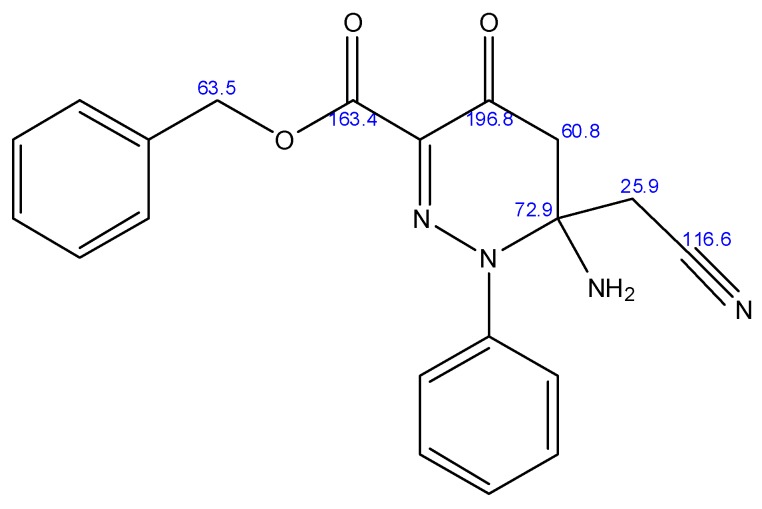
^13^C-NMR spectroscopic analysis of **13a**.

The structures of compounds **13a–c** were confirmed by spectroscopic analysis. Compound **13a** had an m/z value of 362.53. The ^1^H NMR spectrum of **13a** revealed two multiplets for the prochiral CH_2_-CN at δ = 1.28 and 1.31 ppm. A two multiplets for the prochiral ring methylene protons COCH_2_ appears at δ = 2.39 and 2.49 ppm. A singlet at δ = 4.30 ppm is assigned for two methylene protons, a multiplet at δ = 7.10 ppm for the aromatic 10H, and two singlets for the four NH_2_ protons at δ = 11.56 ppm and 14.22 ppm, respectively. The ^13^C-NMR spectrum of compound **13a** showed a signal at δ = 196.8 ppm significant for a true carbonyl, assigned to the pyridazine ring carbonyl, and showed another signal at δ = 163.4 ppm for the ester carbonyl. The CH_2_CN methylene carbon appears at δ = 25.9 ppm. Figure 3 indicates the most important ^13^C-NMR signals for **13a**.

## 3. Materials and Methods 

### 3.1. General

Q-tube assisted reactions were performed in a Q-tube safe pressure reactor from Q Labtech (East Lyme, CT 06333, New London County, CT, USA, equipped with a cap/sleeve, pressure adapter (120 psi), needle adapter/needle, borosilicate glass tube, Teflon septum, and catch bottle. All reactions were monitored by using TLC with 1:1 ethyl acetate-petroleum ether as eluent and were carried out until starting materials were completely consumed. Melting points are reported uncorrected and were determined with a Sanyo (Gallenkamp, Osaka, Japan). ^1^H NMR and ^13^C-NMR spectra were done at the Analab Kuwait University and determined by using a Bruker DPX instrument at 600 MHz for ^1^H-NMR and 150 MHz for ^13^C-NMR and either CDCl_3_ or DMSO-*d*_6_ solutions with TMS as internal standards. Chemical shifts are reported in δ (ppm). Mass spectra and accurate mass measurements were made using a GCMS DFS spectrometer (Thermo, Bremen, Germany) with the EI (70 EV) mode. X-ray crystallographic structure determinations were performed by using Rapid II (Rigaku, Tokyo, Japan) and X8 Prospector (Bruker, Karlsruhe, Germany) single crystal X-ray diffractometers.

### 3.2. General Procedures for Q-Tube-Assisted Synthesis of Quinolines 6**a**,**b**

Glycerol **1** (5 mL), concentrated sulfuric acid (1 mL), and 0.01 mol of the corresponding aniline (0.93 g 3**a** or 1.29 g of 3**b**) were sequentially added in a 35 mL Q-tube pressure tube, furnished by Q Labtech. A Teflon septum was placed on top of the tube, and an appropriate cap was used. The mixture was heated in an oil bath at 200 °C for about 60 min. The mixture was cooled and poured into ice-water. The solid was collected by filtration and purified by column chromatography and crystallized from ethanol.

### 3.3. 6,8-Difloroquinoline 6b

Yellow crystals, yield 60%, mp >250 °C, ^1^H-NMR (DMSO-*d*_6_, 600 MHz): δ = 7.62–7.70 (3H, m, H-3, H-5, H-7), 8.38 (1H, m, H-4), 8.90 (1H, t, H-2). ^13^C-NMR (150 MHz, DMSO-*d*_6_): δ = 105.12, 107.13, 123.37, 129.23, 135.04, 150.16, 156.90, 157.75, 158.61, 159.38. EI-HRMS: *m*/*z* for C_9_H_5_F_2_N, calcd. 165.0390, found: 165.0384.

### 3.4. General Procedure to Aniline Trimers 7 and 8

A 35 mL Q-tube pressure tube, furnished by Q Labtech was charged with aniline derivative **3c-e** (10 mmol) (2.23 g of **3c**, 1.86 g of **3d,** 1.41 g of **3e**), 5 mL glycerol, 3 mL H_2_SO_4_, and 10 mL of water. A Teflon septum was placed on top of the tube, and an appropriate cap was used. The mixture was heated in an oil bath at 160 °C for 15 min. After cooling at room temperature, pH was adjusted at 8–9 by adding NaOH and the reaction mixture was extracted with ethyl acetate (2 × 20 mL). The combined organic layers were dried over MgSO_4_ and were then filtered and evaporated under reduced pressure. The crude residue was purified by column chromatograph (cyclohexane–EtOAc) on silica gel yielding the corresponding quinoline. 

#### 3.4.1. 4,4′,4″-(propane-1,2,3-triyl)tris(2-methylaniline) 7

Yellow crystals yield 75%. mp 178–180 °C, ^1^H-NMR (DMSO-*d*_6_, 600 MHz) : δ = 2.00 (9H,s, 3-CH_3_), 3.34 (5H, m, 2CH_2_ and CH aliphatic), 5.01 (6H, s, 3NH_2_), 6.42 (3H, d, Ar-H), 7.13–7.20 (6H, m, Ar-H), ^13^C-NMR (150 MHz, DMSO-*d*_6_): δ = 16.95 (3CH_3_), 74.0 (CH-aliphatic), 76.24 (2CH_2_-aliphatic), 116.27 (3 *ortho*-C), 124.21 (3C-CH_3_), 134.63 (6 *meta*-C), 137.55 (3*para*-C), 146.53 (3C-NH_2_). EI-HRMS: *m*/*z* for C_24_H_29_N_3_, calcd. 359.2361, found: 359.2362.

#### 3.4.2. 4-(2-(4-amionphenoxy)-3-(4-amoinophenoxy))aniline 8

Brown crystals, yield 72%. mp 240 °C. ^1^H-NMR (DMSO-*d*_6_, 600 MHz): δ = 2.49 (6H, s, 3NH_2_), 3.27 (2H, d, *J* = 6 Hz, CH_2_), 3.29 (2H, d, *J* = 6 Hz, CH_2_), 3.35 (1H, m, CH), 6.94-7.26 (14H, m, Ar-H). ^13^C-NMR (150 MHz, DMSO-*d*_6_): δ = 63.09 (2C, CH_2_), 72.49 (CH), 118.57 (3C, C-NH_2_), 122.01 (2C-O), 129.32 (12C, Ar-C), 140.07 (C-O), EI-HRMS: *m*/*z* for C_21_H_23_N_3_O_3_; calcd. 365.1739; found: 365.1736.

### 3.5. General Procedure for Syntheses of 13**a**–**c**

#### 3.5.1. Method A: Conventional Heating

A mixture of 0.01 mol of the appropriate azo compound **10a**–**c** (**10a**, 2.96 g, **10b**, 2.59 g, **10c**, 2.34 g) is added to **11a** (0.66 g, 0.01 mol) in glycerol (5 mL). The reaction mixture was refluxed for 3–5 h (followed until completion by TLC using 1:1 ethyl acetate- petroleum ether as an eluent). The mixture was cooled and then was poured onto ice-water. The solid, so formed, was collected by filtration and recrystallized from EtOH.

#### 3.5.2. Method B: Q-Tube Assisted Reactions

A 35 mL Q-tube pressure tube, furnished by Q Labtech was charged with 0.01 mol of the appropriate azo compound **10a**–**c** (2.96 g of **10a**, 2.59 g of **10b**, 2.34 g of **10c**), (0.66 g, 0.01 mol) of malononitrile **11a** and 5mL glycerol. A Teflon septum was placed on top of the tube, and an appropriate cap was used. The mixture was heated at 150 °C for 30 min followed until completion by TLC using 1:1 ethyl acetate-petroleum ether as an eluent. The mixture was cooled and then was poured onto ice-water. The solid, so formed, was collected by filtration and recrystallized from EtOH.

### 3.6. Benzyl 6-amino-6-(cyanomethyl)-4-oxo-1-phenyl-1,4,5,6-tetrahydropyridazine-3-carboxylate *13***a**

Yellow crystals, yield 52% (method A), 76% (method B); mp >340 °C. ^1^H-NMR (DMSO-*d*_6_, 600 MHz): δ = 1.28,1.31 (2H, m, CH_2_), 2.39,2.49 (2H, CH_2_, m), 4.30 (2H, s, CH_2_), 7.10–7.53 (10H, m, 2-Ph), 11.56, 14.22 (2H, 2s, NH_2_). ^13^C-NMR (150 MHz, DMSO-*d*_6_): δ = 196.8 (C=O), 164.5 (C=O), 163.0 (N-C-Ar), 142.8 (N=C), 132.0 (2C-Ar), 131.2 (3C-Ar), 129.8 (2C-Ar), 125.8 (2C-Ar), 116.6 (CN), 115.6 (2C-Ar), 72.9 (C-NH_2_), 60.8 (CH_2_-O), 30.8 (CH_2_), 25.9 (CH_2_). HRMS: *m*/*z* (EI) for C_20_H_18_N_4_O_3_, calcd. 362.1379, found: MS: *m*/*z* (%) = 362.53 (M^+^).

### 3.7. Ethyl-6-amino-5-cyano-6-(cyanomethyl)-4-oxo-1-phenyl-1,4,5,6-tetrahydropyridazine-3-carboxylate 13b

Red crystals, yield 64% (method A), 88% (method B); m.p. 256-258 °C, IR (KBr): 3425 (NH), 2202 (CN), 1620 (CO) cm^−1^, ^1^H-NMR (DMSO-*d*_6_, 600 MHz): δ = 1.04 (3H,t, CH_3_), 1.05 (2H,m, CH_2_), 2.03 (1H,s, CH), 3.33 (2H,s, CH_2_), 4.32 (2H,m, CH_2_), 7.27-7.64 (5H, m, H-Ph). ^13^C-NMR (150 MHz, DMSO-*d*_6_)): δ = 13.90 (CH_3_), 24.0 (C-CN), 26.69 (CH_2_), 61.23 (C-O), 66.0 (C-NH_2_), 115.90 (2C; 2CN), 119.21 (2C-Ar), 123.87 (C-Ar), 128.88 (2C-Ar), 129.51 (C=N), 138.34 (C-Ar), 160.97 (2C=O). MS (EI): *m*/*z* = 326.0 (M^+^). HRMS: *m*/*z* (EI) for C_16_H_15_N_5_O_3_, calcd. 325.1175, found: MS: *m*/*z* (%) = 326.0 (M^+^).

### 3.8. Ethyl-6-amino-6-(2-ethoxy-2-oxoethyl)-4-oxo-1-phenyl-1,4,5,6-tetrahydropyridazine-3-carboxylate 13c

Brown crystals, yield 70% (method A), 92% (method B), m.p. 266–268 °C, IR (KBr): 3439 (NH), 2222 (CN), 1670 (CO) cm^−1^, ^1^H-NMR (DMSO-*d*_6_, 600 MHz) : δ = 1.20 (3H,t, CH_3_), 1.40 (3H,t, CH_3_), 2.50 (2H,s, CH_2_), 3.36 (2H,s, CH_2_), 4.10 (2H,m, CH_2_), 4.35 (2H,m, CH_2_), 7.07-7.55 (5H, m, H-Ph), 10.29 (1H, s, NH). ^13^C-NMR (150 MHz, DMSO-*d*_6_): δ = 8.37 (2C- CH_3_), 62.59 (CH_2_), 63.06 (CH_2_), 66.24 (CH_2_-O), 68.81 (CH_2_-O), 72.48 (C-NH_2_), 114.96 (C-Ar), 116.15 (CN), 116.30 (C-Ar), 123.62 (C-Ar), 129.05 (C-Ar), 129.62 (C-Ar), 130.16 (C=N), 142.42 (C=O), 161.72 (C=O), 196.22 (C=O). HRMS: *m*/*z* (EI) for C_17_H_21_N_3_O_5_: 347.1481, found: MS: *m*/*z* (%) = 347.1 (M^+^).

## 4. Conclusions

In this study, we combined glycerol and water as green, efficient solvents, in addition to pressure in Q-tubes, to modify the synthesis of quinolines and prepare new aniline trimers and pyridazines. Glycerol was efficiently employed either as a catalyst or a reactant and green bio-based solvent in the synthesis of novel quinolines, aniline trimers, and pyridazines. The dual use of glycerol along with reactions under pressure proved its efficiency as a green method for synthesis of quinolines as reactions products were obtained in higher yields, shorter time, and without any oxidizing agents. Reactions of anilines, sulphuric acid, water, and glycerol under pressure allowed for the synthesis of unexpected novel aniline trimers. Lastly, glycerol also proved to be an efficient medium/catalyst for synthesis of novel pyridazines in very good yields under pressure. Future perspectives for this work are various since our techniques should open the appetite of researchers to extend this work for the synthesis of azoles, azines, and other polyanilines.

## Figures and Tables

**Table 1:** Variation of the nature of aniline products depending on the reaction conditions in the presence and absence of H_2_O, starting from glycerol **1** under a high pressure, and high temperatures in the Q-tubes.

Entry	R_1_	R_2_	R_3_	Reaction Conditions			Product	Yield%	MP. (°C)	*m*/*z*
				Medium	Time (min.)	Temp (°C)				
**3a**	H	H	H	glycerol	60	200	6a	58	BP = 238	
**3a**	H	H	H	glycerol + H_2_O	15	160	8	73	240	365.17
**3b**	F	F	H	glycerol	60	200	6b	60	243	165.03
**3c**	CH_3_	I	H	glycerol + H_2_O	15	160	7	75	180	359.23
**3d**	CH_3_	Br	H	glycerol + H_2_O	20	160	7	70	180	359.23
**3e**	CH_3_	Cl	H	glycerol + H_2_O	20	160	7	70	180	359.23
**3f**	H	H	Cl	glycerol + H_2_O	30	160	not concluded	-		410

**Table 2:** Reaction yield for **13a–c** and **12d** under pressure and conventional heating.

Product	x	y	z	Yield Percentage	
				Conventional Heating	Under Pressure
**13a**	CN	COOCH_2_Ph	H	52	76
**13b**	CN	COOEt	CN	64	88
**13c**	COOEt	COOEt	H	70	92
**12d**	CN	H	CH_3_	86.16 [74]	-
